# Understanding “Internet Plus Healthcare” in China: Policy Text Analysis

**DOI:** 10.2196/23779

**Published:** 2021-07-26

**Authors:** Feng Yang, Huilin Shu, Xiaoqian Zhang

**Affiliations:** 1 Sichuan University Chengdu China; 2 McGill University Montreal, QC Canada

**Keywords:** internet plus health care, China, policy analysis, COVID-19, epidemic

## Abstract

**Background:**

The combination of the internet and healthcare has excellent benefits and far-reaching positive effects in improving service efficiency and promoting social equity. The role of the “internet plus healthcare” (IPHC) has been recognized, especially during the COVID-19 pandemic. This new healthcare model is also familiar to people and shows a bright prospect.

**Objective:**

This article seeks to accurately understand and fully grasp the characteristics of IPHC policies that can enlighten the formulation of future policies.

**Methods:**

The content analysis method was used to analyze China’s IPHC policies collected from the Beida Fabao database and several official websites.

**Results:**

We found that the development of IPHC policy has gone through 4 stages and is currently entering a phase of rapid development. IPHC policymakers are primarily health administrative departments. In addition, policy instruments are classified into either supply, environment, or demand, and policy themes can be summarized into 4 categories: facilities, technology, service, and management.

**Conclusions:**

China’s IPHC policy has good prospects from the perspective of development trends. The health administrative departments mainly lead the development of China’s IPHC policy. It is suggested that these departments involve other stakeholders (ie, medical workers, medical industries, and technology sectors) in formulating policies. Policies prefer to use supply-based and environment-based policy instruments. The policy themes emphasize improving infrastructure construction and high-quality diagnostic and treatment services, strengthening the supporting role of information technology, and ensuring all stakeholders understand their responsibilities.

## Introduction

The internet has become a driving force for different fields, including healthcare, education, and entertainment. To maximize the potential of the internet, the Chinese government has created “internet plus” designs to transform, modernize, and equip traditional industries. Likewise, the healthcare industry has been combined with the internet to create “internet plus healthcare” (IPHC).

IPHC is a novel application of the internet in the healthcare industry that includes health education, medical information queries, electronic health records or electronic medical records (EHR/EMRs), disease risk assessments, online disease consultations, electronic prescriptions, remote consultations, and various remote forms of health and medical services such as treatment and rehabilitation. Chinese hospitals use the internet and mobile technologies to alleviate the challenges patients encounter when obtaining hospital services [1]. Since the outbreak of COVID-19, IPHC has played a role in “high efficiency and low risk” health care delivery [2] by enabling a more efficient pandemic response and launching a variety of valuable services such as addressing pandemic queries in real-time, facilitating online consultations, and providing home isolation guidelines. IPHC also minimizes the risk of cross-infection via in-person consultations. As a result, the IPHC model underwent significant development in 2020. For example, Alibaba Health launched a free clinic service on the internet, and Jingdong Health launched the “Preventing and Blocking COVID-19 Pneumonia” platform.

The development of IPHC relies on effective national policies. Policies reflect conceptualization, subjectivity, and practice in specific fields. Policy analysis enables an understanding of previous policies and offers retrospective and prospective insights for future policy development and implementation [3]. It offers a robust path to understanding how and why governments enact certain policies, including their values, interests, and the political contexts [4]. As a solid reflection of public affairs and the essence of policy content, policy text has become the primary starting point for policy analysis. Converting the policy text into several elements allows for a better examination of themes contained in the policy. Health policy analysis can potentially resolve protracted policy disputes and strengthen a sustainable health system [5].

Existing research on health policy in China either focuses on reformation [6-8] or tries to assess how specific diseases affect China’s health policy [9,10]. Previous studies evaluated the combination of the internet and healthcare in China by studying internet hospitals. For example, Xie et al [11] provided an overview of the internet hospitals in China; Han et al [12] analyzed the construction and content of internet hospitals in China. In addition, the number of Chinese citizens who use the internet to seek health services is increasing; therefore, there is an urgent need to update and refine the current IPHC policies [13]. Several health policy analysis frameworks have been proposed, including the policy triangle framework [14] and the network frameworks [15]. Considering “researchers need to use existing frameworks and theories of the public policy process more extensively [3],” the analysis of IPHC policies will combine the policy triangle framework and the network frameworks with practical requirements. This paper will address the following research questions, which contain elements proposed by previous research and can be collected from policy texts:

1. When are these policies issued?
2. Who issues these policies?
3. What policy instruments are included in these policies?
4. What are these policy themes?

By answering these questions, this paper aims to track the policy trajectory of IPHC in China, present the instruments guaranteed for policy implementation, and understand the key themes of IPHC.

## Methods

### Research Materials

Policy text was collected in 3 steps from several sources published on May 10, 2020. First, we used the Beida Fabao database, one of China’s most professional legal databases, characterized by rich content, detailed classification, and timely data. The retrieval strategy was to obtain relevant policy text with keywords, including “internet medical,” “network medical,” “internet health,” and “network health” through a full-text search. Second, we retrieved new keywords in the obtained policy text, such as “electronic health records,” “telemedicine,” and “internet hospital,” as clearly stated in the “Opinions of the General Office of the State Council on Promoting the Development of ‘Internet plus Healthcare.’” Finally, we supplemented the retrieval strategy with policy text published on the official websites of affiliated agencies such as the National Health Commission of the People’s Republic of China (NHC) and the National Administration of Traditional Chinese Medicine (SATCM). Given that the content of local IPHC policies was mainly based on national policies, and the focus of local policy themes was consistent with national policies, IPHC policies issued by the local government were eliminated from the analysis. Likewise, duplicate policy and invalid policy documents were removed, resulting in 90 policy texts.

### Research Method

The content analysis method was used to convert a large amount of text into a small number of categories to help researchers discover the policy development patterns and trends [16]. The content analysis was designed to examine the text and explain “what they mean to people, what they enable or prevent, and what the information conveyed by them does” [17]. The content analysis method identified IPHC policy codes and classified the textual information per the following criteria:

#### The Evolution of Policies

The issuance period shows the development trend of the IPHC policies. We coded the date of policy issuance into annual units.

#### Policymakers

The coding for issuing markers to government agencies enables us to distinguish which government agencies are mainly involved in policymaking and which government agencies cooperate more in the policymaking process. We coded the policymakers by collecting their names as written in the policy issuing agencies sections of the policy texts. Then we standardized the names of policymakers according to the latest 2018 State Council Institutional Reform Plan.

#### Policy Instruments

Based on the classification of policy instruments by Rothwell and Zegveld [18], policy instruments in IPHC policies were classified into supply-based (eg, “technology and infrastructure” and “education and training”), environment-based (eg, “goal planning” and “legal supervision”), and demand-based policy instruments (eg, “medical insurance system” and “online services”). For example, the “Notice of the State Administration of Traditional Chinese Medicine on Printing and Distributing the Twelfth Five-Year Plan for the Informatization of Traditional Chinese Medicine” can be coded as “integrated medical,” “information support,” “internet-based Chinese medicine,” and “education and training.” According to this classification, 2 coders coded the 90 policy texts.

#### Policy Themes

Policy themes can reflect the significant concerns derived from policy documents. We retrieved keyword segmentation results using the Jieba for Python and analyzed high-frequency keywords. We formulated the co-occurrence matrix of high-frequency keywords to examine the main clusters of IPHC themes from the textual information. We used Gephi to visualize the policy themes.

The consistency of the coding results by 2 coders was tested to ensure the reliability and objectivity of the content analysis of IPHC policy documents. The formula which was used to determine reliability was consistency coefficient = 
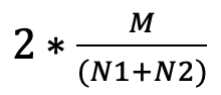
 (where M is the number of consistent codes derived from the 2 coders, and N1 and N2 are the coder’s coding numbers). The calculated result showed that the consistency coefficient of coding was within an acceptable range (0.854).

## Results

### The Evolution of Policies

**Figure 1:**
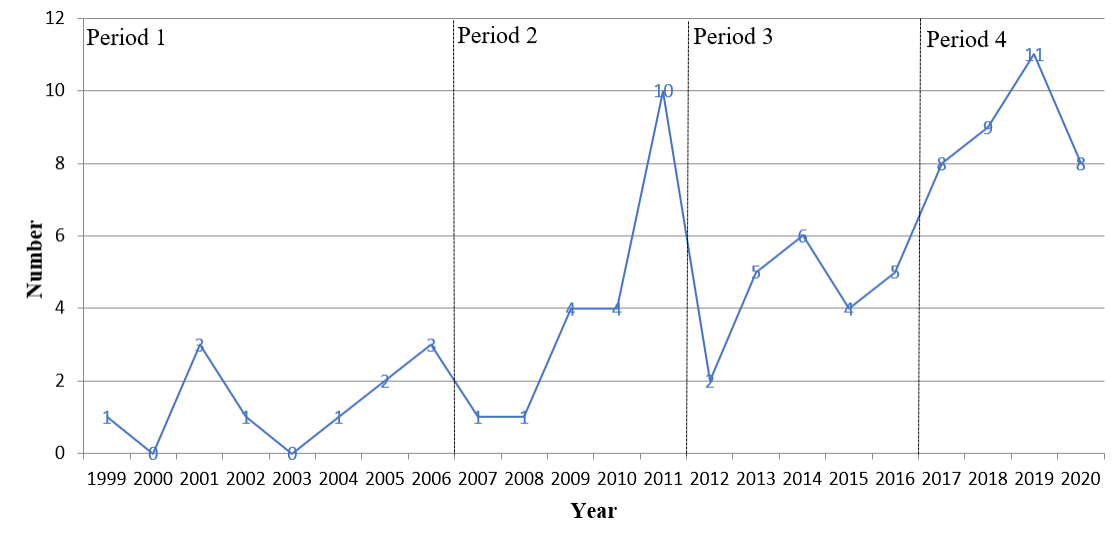
Time distribution of “internet plus healthcare” policy.

As an essential part of China’s national economic plan, The Five-Year Plan makes long-term plans for China’s major construction projects. It specifies the goals and directions for national economic development. According to the 5-year plans for the development of health services issued by the State Council (SC; “Notice of the State Council on Approving and Transmitting the Outline of the ‘Eleventh Five-Year Plan’ for the Development of Health Services,” on May 21, 2007; “Notice of the State Council on Printing and Distributing the ‘Twelfth Five-Year Plan’ for the Development of Health Services,” on October 8, 2012; and “Notice of the State Council on Printing and Distributing the ‘Thirteenth Five-Year’ Health and Wellness Plan,” on December 27, 2016), the development of IPHC policy can be divided into four stages (Figure 1), namely, “The Budding Period” (1999-2006), “The Initial Development Period” (2007-2011), “The Reform and Development Period” (2012-2016), and “The Rapid Development Period” (2017-present).

### Policymakers

The results of IPHC policymakers are shown in Table 1. Results showed that 24 departments issued IPHC policies, including NHC, SATCM, National Medical Products Administration (NMPA), and National Development and Reform Commission. The NHC is the leading organization and has published the most policies (49 policies). This is because the main responsibility of the NHC is to organize the formulation of national health policies, laws, regulations, plans for health development, and departmental rules and standards and organize their implementation. Of the 90 IPHC policy texts, 73 (81.11%) policies were promulgated independently by 1 department. There are 17 joint promulgated policies, and the NHC participates in each promulgated joint policy. The 15 departments represented by The State Council Information Office of the People’s Republic of China, Ministry of Education of the People’s Republic of China (MOE), and Ministry of Finance of the People’s Republic of China (MOF) have promulgated one policy each, all of which are issued jointly.

**Table 1:** Statistics of each IPHC policy-issuing department.

Policymakers	Number of policies	Joint number of policies	Frequency of joint policies (%)
National Health Commission of the People’s Republic of China	49	17	34.69
National Administration of Traditional Chinese Medicine	27	9	40.91
National Medical Products Administration	19	2	7.14
National Development and Reform Commission	4	4	100
State Council	4	0	0.00
Ministry of Industry and Information Technology of the People’s Republic of China	3	3	100
National Healthcare Security Administration	3	2	66.67
Ministry of Science and Technology of the People’s Republic of China	3	3	100
Ministry of Civil Affairs of the People’s Republic of China	2	2	100
The State Council Information Office of the People’s Republic of China	1	1	100
Ministry of Education of the People’s Republic of China	1	1	100
Ministry of Finance of the People’s Republic of China	1	1	100
Ministry of Human Resources and Social Security of the People’s Republic of China	1	1	100
Ministry of Natural Resources of the People’s Republic of China	1	1	100
Ministry of Ecology and Environment of the People’s Republic of China	1	1	100
Ministry of Housing and Urban-Rural Development of the People’s Republic of China	1	1	100
Ministry of Commerce of the People’s Republic of China	1	1	100
Ministry of Culture and Tourism of the People’s Republic of China	1	1	100
The People’s Bank of China	1	1	100
State Taxation Administration of the People’s Republic of China	1	1	100
State Administration for Market Regulation	1	1	100
General Administration of Sport of China	1	1	100
China Banking and Insurance Regulatory Commission	1	1	100
China Securities Regulatory Commission	1	1	100

### Policy Instruments

Policy instruments reflect the intent of policy formulation and determine its effectiveness. They are selected based on economic, political, and social contexts [19], but they are rarely mentioned in public health literature [20]. This study refers to the classification of policy instruments by Rothwell and Zegveld [18], which includes supply-based policy instruments (public enterprise, scientific and technical, education, and information), demand-based policy instruments (procurement, public service, commercial, and overseas agents), and environment-based policy instruments (political, legal and regulation, taxation, and financial). The constructed analysis framework of the IPHC policy instruments is shown in Textbox 1. Supply-based instruments ensure the government directly supports various stakeholders with technology, infrastructure, talent, and resources, thereby promoting IPHC development. Environment-based policy instruments create a suitable environment for developing the IPHC industry by improving laws and regulations and supervisory protocols with clear inspection systems. Finally, demand-based policy instruments explore the demand for IPHC services and promote its development through the medical insurance system and online services.

Results of statistics on the number of policies of various types of IPHC policy instruments are shown in Table 2. Overall, the different types of policy instruments are involved. However, supply-based and environment-based policy instruments are used more, while demand-based policy instruments are used less. Among the supply-based policy instruments, the most used is the “information support,” accounting for 47.78% of the 90 policies, followed by the “management” and “legal supervision,” which account for 38.89% and 35.56% of environment-based policy instruments, respectively. The least used policy instrument is the “performance assessment” (8.89%).

**Analysis framework of IPHC policy instruments.**

**Supply-based**

- Technology and infrastructure: Provide the necessary infrastructure and technology to develop IPHC.
- Education and training: Carry out various education and training activities for practitioners involved in IPHC, provide learning resources, and strengthen the development of relevant talents.
- Information support: Build related databases and knowledge bases and make full use of information technologies to provide information exchange and information services to develop IPHC.
- Resource allocation: Medical resources, financial subsidies, and other resources are allocated according to the characteristics and needs of the development of IPHC.

**Environment-based**

- Goal planning: The development direction of IPHC is defined by formulating macro targets and overall planning.
- Legal supervision: IPHC stakeholders are regulated by establishing regulations, laws, and industry standards.
- Management: The quality of services or products provided by medical and health institutions and related enterprises is managed.
- Policy publicity: IPHC policy is publicized actively to expand the beneficiary groups of the policy and generate a positive public opinion.
- Entities collaboration: The relevant entities of IPHC are encouraged to collaborate and actively promote IPHC development.
- Performance assessment: A scientific performance assessment system is given to subjects offering IPHC information and services.

**Demand-based**

- Medical insurance system: Users’ rights and interests in IPHC activities are fully protected through continuous improvement and optimization of the medical insurance system.
- Online services: Traditional offline consultation and sales services are transformed into convenient and reliable online services through internet technology platforms.
- Integrated medical: Users are offered a more convenient medical experience through the interconnection of medical and health information resources and the full integration of high-quality medical resources.
- Internet-based Chinese medicine: The integrated development of Chinese medicine health service and the internet should be promoted.

**Table 2:** Statistics of IPHC policy instruments.

Policy instruments		Number		Frequency (%)
**Supply-based**				
	Technology and infrastructure	27	30.00	
	Education and training	18	20.00	
	Information support	3	47.78	
	Resource allocation	11	12.22	
**Environment-based**				
	Goal planning	15	16.67	
	Legal supervision	32	35.56	
	Management	35	38.89	
	Policy publicity	11	12.22	
	Entities collaboration	18	20.00	
	Performance assessment	8	8.89	
**Demand-based**				
	Medical insurance system	12	13.33	
	Online services	16	17.78	
	Integrated medical	21	23.33	
	Internet-based Chinese medicine	9	10.00	

### Policy Themes

**Figure 2:**
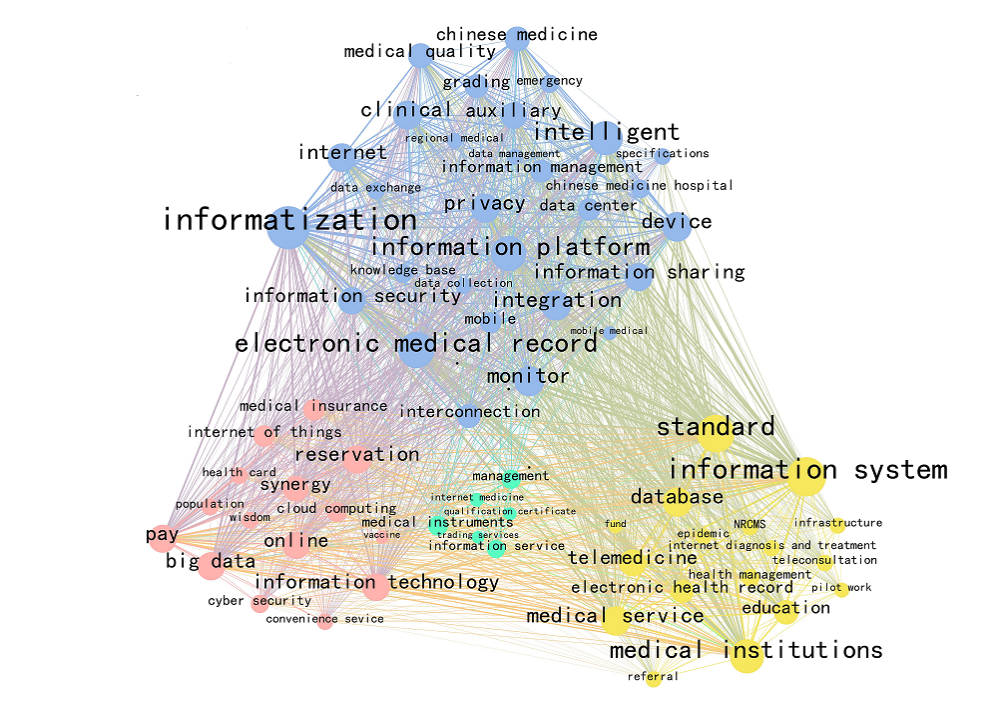
The visualization map of high-frequency keywords.

A total of 4586 keywords were extracted using Jieba for Python. The *T* value was approximately 37 based on the high-low frequency words boundary fraction formula 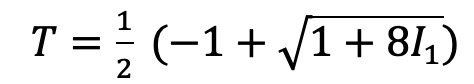 proposed by Donohue [21], where *T* is the lowest frequency among the high-frequency keywords, and *I*_1_ is the number of keywords with high frequency. There were 1107 keywords with a frequency greater than 37, and a visualization map with high-frequency words was generated using Gephi. Gephi is a data visualization software that can cluster points of the same attribute (Figure 2). As a result, the themes of IPHC policy can be summarized into 4 categories: facilities (blue), technology (pink), services (yellow), and management (green).

## Discussion

### Principal Findings

The study found that IPHC policy can be divided into 4 phases: “The Budding Period” (1999-2006), “The Initial Development Period” (2007-2011), “The Reform and Development Period” (2012-2016), and “The Rapid Development Period” (2017-present). In addition, IPHC policymakers are led by the health administrative department. As the core department, the NHC has formulated the most policies and cooperates extensively with MOE, MOF, and other departments. Furthermore, China’s IPHC policy instruments cover 3 types: supply, environment, and demand, with a preference for the supply-based and environment-based policy instruments. Finally, IPHC policy themes can be summarized into 4 categories: facilities, technology, services, and management, emphasizing the improvement of infrastructure construction, strengthening the supporting role of information technology, improving high-quality diagnosis and treatment services, and ensuring all stakeholders understand their responsibilities.

### The Prospect of Policy Development

An early prototype of China’s IPHC appeared in the 1990s when doctors in China began to communicate with experts in other countries through emails about clinically incurable diseases. After that, with the increasing use of computers for remote medical consultations in various places, NHC issued the “Notice on Strengthening the Management of Telemedicine Consultation” in 1999 to regulate medical order and medical behavior and enable the development of healthy and orderly telemedicine consultation work. However, general interest in information technology and internet use among the public was limited at that time. Thus, the number of related policies released during that period was relatively small. At this stage, the largest number of policies was promulgated in 2001. The 3 policies issued in 2001 all focused on the “Internet Drug Information Service” and the regulation of drugs obtained online.

With the innovation of information technology and the widespread popularity of information networks, SATCM issued the “Outline of the Eleventh Five-Year Plan for Chinese Medicine Informatization” in 2007, emphasizing the construction of various information systems in healthcare. At the end of 2010, NHC issued the “Electronic Medical Record System Functional Specification (Trial),” which regulated the management of EHR/EMRs in medical institutions, promoted the construction of hospital informatization, and profoundly affected subsequent policy development. For example, the number of policies issued in 2011 peaked at this stage, and most of them emphasized the construction of hospital informatization based on EHR/EMRs. Overall, the integration of healthcare and the internet ushered in a preliminary development stage. As a result, the relevant policies issued were further improved, with policies covering a more comprehensive range of topics. In addition, the basic information database was completed, the medical management information system was continuously improved, and talent training programs in the medical field were initiated.

In 2011, many medical institutions and doctors began to use Weibo (a popular social media app in China) to interact with patients. However, with the ensuing privacy exposure and medical disputes, government departments began to regulate and manage social media–based medical services, and IPHC entered a period of deepening reform. Overall, more policies were issued at this stage compared to the previous stage, and the number of policies fluctuated less frequently. Various targeted measures were introduced to IPHC to resolve issues regarding an imperfect management system and operation mechanism, the lack of information infrastructure development, low levels of information technology application, and the insufficient sharing of medical information resources. For example, specific policies have been issued for telemedicine services, information system development, information technology training, and internet pharmaceutical sales. In addition, the Communist Party of China (CPC) and the SC jointly issued “Healthy China 2030” in 2016, which outlines the blueprint and action plan for the construction of a healthy China over the next 15 years, proposing to regulate and promote IPHC services, and innovate the IPHC service model. The introduction of this outline was the first time that the country clearly stated its attitude toward IPHC and elevated it to the level of a national strategy.

With the reform and development of the previous stage, China’s healthcare has made significant progress, and people’s health has continued to improve. Therefore, the CPC and SC have attached great importance to the development of health and wellness and placed it in a prominent position for economic and social development. In the 19th National Congress of the CPC report on October 18, 2017, the development strategy of “healthy China” was put forward, and China’s IPHC policy entered a period of rapid development. In 2018, in response to the problems encountered in the vigorous development of IPHC, the General Office of the SC issued “Opinions on Promoting the Development of ‘internet plus healthcare,’” proposing a series of policies to promote the integration of the internet and medical health and urge all departments to issue supporting policy measures promptly. Furthermore, as a result of COVID-19, the NHC successively issued several departmental regulatory documents before May 2020 to leverage the complete advantages of internet healthcare, which has provided robust support for epidemic prevention and control. As such, the pandemic has promoted the development of IPHC to a certain extent, and the number of related policies is expected to increase significantly in the near future.

### The Multiple Voices in Policymaking

The “internet plus” industry, including IPHC, is policy-oriented in China. Compared to other industries, the IPHC field is more professional and requires complete, detailed, and operable policy documents to regulate industry behavior and guide industry development. Overall, the current IPHC policy issuance is mainly led by the health administrative department. For example, the NMPA is responsible for internet-based drug-related policies, and the NHC coordinates industry development and formulates industry standards. In addition to continuing its primary role in future policy formulation, it strengthens the coordination with nonhealthy departments to leverage the advantages of each department. It is suggested that these departments should be involved in formulating policies for various social services. They should listen to the voices of different interest groups in the IPHC field, coordinate conflicts of interest, and enhance the enforceability of policies. For example, medical personnel and the medical industry offer a unique perspective in formulating health policies because of their knowledge, technology, and position [3]. In addition, the health administration should strengthen communication and cooperation with internet companies, telecommunications industries, communities, and the general public.

### The Coordination of Various Policy Instruments

“Technology and infrastructure” account for a relatively high proportion (30%) of supply-based policies; however, the importance of such policies on emerging technologies needs to be improved. Nambisan [22] found healthcare organizations that provide patients with online health communities need to pay more attention to developing tools that will make internet searches more effective. Considering the role of cloud computing, big data, artificial intelligence, 5G, and other emerging technologies in promoting the positive development of IPHC, future policies need to closely follow technology development trends and provide forward-looking and innovative guidance for the development of IPHC.

“Education and training” accounts for 20% of all policy instruments and can be divided into 2 categories: continuing education for medical practitioners and providing a training program for the new personnel joining the IPHC industry. Compared to the former, the latter has had a late start, and its policy content is primarily macro; thus, it is necessary to provide detailed training programs for new talent. The number of policies using “information support” covers the largest proportion (47.78%) of supply-based policy instruments. They all emphasize improving the level and ability of medical information services and actively creating a development culture for the growth of IPHC through the construction of information platforms and other informatization means. The “resource allocation” policy instruments are used less frequently, accounting for only 12.22%; hence, future policies should consider further strengthening financial investments.

Among the environment-based policy instruments, “management” is used the most (39.89%). It is mainly focused on EHR/EMRs, telemedicine, and internet drug information services. The least used is “performance assessment” (8.89%) and is reflected in telemedicine pilots, informatization of traditional Chinese medicine and vaccines, and electronic registration after 2015. This type of policy instrument needs to be improved further to provide a scientific performance system to assess the management of relevant practitioners. “Entities collaboration” accounts for 20% of environment-based policy instruments, mainly emphasizing the coordination of supervision among policy issuers and the cooperation of medical institutions; however, participation and collaboration among other social services are relatively neglected. The policies using “policy publicity” account for 12.22% of environment-based policies. Most of them were published after the “Guiding Opinions of the General Office of the State Council on Promoting and Regulating the Application of Health and Medical Big Data” was issued by the General Office of SC in 2016. This policy instrument needs to be used continuously to create a suitable environment for engaging public opinion in developing IPHC.

“Integrated medical” (23.33%) accounts for a large proportion of demand-based policy instruments, and it was developed after 2014. The second is “online services” (17.78%), which emphasized the development of “internet plus diagnosis and treatment” and “internet plus pharmaceuticals,” the interconnection of medical resources, and the optimization of the medical experience. Only 13.33% of the demand-based policy instruments cover the “medical insurance system,” which is essential to protecting people’s health. In addition to the previous medical insurance payment guidance on telemedicine, other policies have begun to use the “medical insurance system” following the “Notice of the General Office of the National Health and Family Planning Commission on the Comprehensive Promotion of the Construction of the National New Rural Cooperative Medical Information Platform” issued in 2015. This policy instrument needs to be improved in terms of pricing instructions, pricing standards, and service areas to effectively solve user needs, dispel user concerns, and promote the development of IPHC. “Internet-based Chinese medicine” accounts for 10% of all demand-based policies, emphasizing the development of the internet plus Chinese medicine health services; however, specific and feasible solutions are still under discussion.

### The Four Categories of the Policy Themes

“Facilities” focuses on the infrastructure (eg, the construction and improvement of network equipment, network environment, databases, information systems, and mobile devices) required during the development of IPHC, utilizing core keywords such as “informatization” and “electronic medical records.” “Informatization is a relatively abstract concept related to the combination of medical treatment and information technology, using information technologies such as the internet to improve medical efficiency and service levels. It is referred to in the policy as “implement a national health security information project, expand and improve existing facilities, fully build a shared population health information platform, and strengthen data collection, integrated sharing, and business collaboration of application information systems such as public health, medical services, and drug supply” [23]. For example, relying on China Unicom’s 5G plus a medical cloud platform, experts in Beijing, Shanghai, Guangzhou, and other places remotely consulted with critically ill patients in Leishenshan Hospital in Wuhan in February 2020, improving medical efficiency. “Electronic medical record” and “informatization” function in tandem. Existing policies emphasize constructing a basic database with EHR/EMRs as the core of healthcare informatization. They also recommend enhancing the unification and standardization of EHR/EMRs standards to achieve big data management of health information for the entire life cycle of the population. However, EHR/EMRs have not been widely popularized in Chinese hospitals. Traditional paper medical records are still the standard, and the transmission and sharing of EHR/EMRs between hospitals are even more limited. As the foundation of IPHC, the optimization of “facilities” is of vital importance, and China still needs to invest additional efforts to ensure the development of IPHC by improving the network, database, and mobile devices.

The evolution of technology can bring substantial benefits to healthcare [24]; even “policymakers worldwide view information technology as a means of making healthcare systems safer, more affordable, and more accessible” [25]. The “technology” theme of China’s IPHC also reiterates the foundational importance of information technology, emphasizing the development of cloud computing, the internet of things, and other information technologies to provide technical support for the development of IPHC. Representative high-frequency keywords in this category include “payment,” “online,” and “synergy.” Among them, “payment” refers to the development of mobile payment technologies (eg, network payment or online payment) to reduce patients’ queuing time and optimize hospital service processes. Especially in recent years, mobile payment terminals have become standard equipment in various hospitals, and patients also tend to use mobile apps such as Alipay and WeChat to pay their fees. “Online” emphasizes the conversion of traditional medical treatment processes to online platforms, including online queries, online appointments, online viewing of medical results, online payment, and online drug purchases. At present, many hospitals have initiated their official portals, WeChat public accounts, and other network portals. By visiting these portals, patients can complete the entire process of scheduling appointments, medical treatments, and medication purchases. Even under the influence of this trend, professional internet medical platforms such as Ping An Good Doctor (PAGD), Ding Xiang, Chunyu, and Good Doctor have begun to appear. For example, Ping An Health Cloud Company launched PAGD (an online health consultation and health management app) in 2015, and the number of user registrations exceeded 315 million by the end of 2019 [26]. Unlike traditional hospitals, PAGD provides users with one-stop medical services, including 24/7 online consultations, referrals, registrations, online drug purchases, and 1-hour drug deliveries. “Synergy” is a collective term for “regional synergy,” “online and offline synergy,” “business synergy,” etc. It is believed that the process of developing IPHC should (1) improve coordination between different regions to promote access to high-quality medical resources in remote areas; (2) optimize the medical service process by coordinating the traditional medical model and online medical model; and (3) coordinate various business-related information, promote the integration of information resources (eg, medical service prices, drug information, and medical insurance payments) and promote the joint reform of medical industry, pharmaceutical industry, and medical insurance system.

The “service” category includes keywords such as “information system,” “medical institution,” and “medical service,” emphasizing the optimization of medical services and the improvement of service levels. Generally speaking, companies can accurately identify consumer needs and quickly integrate resources through the internet to improve services. For hospitals, the use of the internet can provide people with safe, effective, convenient, and inexpensive essential medical services, significantly improve patients’ experience with doctor visits, and improve the quality of medical services. As a result, each medical institution should strengthen the use of information technology, and government departments should improve relevant information and technology standards and strengthen medical information education. For example, several policies promulgated during the COVID-19 epidemic have recognized the role of IPHC in responding to sudden infectious diseases, requiring government departments and hospitals at all levels to leverage the beneficial experience of internet-based diagnosis and treatment, the construction of internet hospitals, and the use of telemedicine services during the pandemic. This further promotes the integrated development of internet technology and medical services and the normalization of telemedicine services.

In developing IPHC, problems such as user privacy, qualifications of practitioners, and responsibilities and obligations of both doctors and patients have become prominent. Improper management of these concerns will risk patients’ health. At the same time, it will stigmatize IPHC and damage the development of the industry. For example, Bansal et al [27] found that users’ trust in medical websites and concerns about privacy affect the extent to which they provide personal health information on the internet, which affects the success of IPHC Therefore, the “management” of patient privacy concerns is necessary. It focuses on the supervision of all stages in the development process of IPHC and specifies the responsibilities of all parties. For example, health administrative departments at all levels should strengthen the supervision and management of internet diagnosis and treatment services, publicize the list of medical institutions that conduct internet diagnosis and treatment activities, and facilitate the supervision of these institutions by the general public.

Furthermore, medical institutions should strengthen the use and management of internet diagnosis and treatment activities, ensure that they are traceable throughout the process, and open data interfaces with regulatory authorities. In addition, physicians who carry out internet diagnosis and treatment activities shall obtain corresponding qualifications according to the law. Currently, government departments at all levels in China have successfully issued specific policies to regulate the development of this industry. For instance, the “Opinions on Promoting the Development of ‘Internet plus Healthcare’” issued in 2018 by the SC required the NHC, Cyberspace Administration of China, Ministry of Industry and Information Technology of the People’s Republic of China, and other departments to issue management measures to regulate internet diagnosis and treatment, to clarify the standards for supervision, and to ensure the quality and safety of medical and health services. Likewise, the Sichuan Province, Zhejiang Province, and other places have also established provincial level IPHC supervision platforms. These platforms supervise online medical institutions, prescriptions, diagnosis and treatment content, service quality, and the qualifications of doctors, nurses, and pharmacists.

### Conclusions

IPHC policy clarifies the development goals of the medical industry and the main contents of industry development. It also promotes the modernization of medical service methods. This article used content analysis to analyze policymakers, policy instruments, and the policy themes of IPHC policy in China, aiming to determine the characteristics of current policies, examine the inherent laws of policies, inspire future policy formulation, and guide the policies of the IPHC industry better.

This article has some limitations. Considering that the local policies were issued under the guidance of the central policy, we only selected the policies at the central level for analysis. However, the study of local policies can effectively show the spread, speed, and influence of policies and identify regional characteristics for future research. In addition, although this article analyzes several aspects of IPHC policy, it is limited by time and effort and cannot further explain the interconnection between these aspects. For example, which policymaker prefers to use which policy instrument, and do different policy themes use other policy instruments? Additional in-depth research is required to answer these questions.

## Abbreviations

CPC: The Communist Party of China
EHR/EMR: electronic health record or electronic medical record
IPHC: internet plus healthcare
MOE: Ministry of Education of the People’s Republic of China
MOF: Ministry of Finance of the People’s Republic of China
NHC: The National Health Commission of the People’s Republic of China
NMPA: National Medical Products Administration
PAGD: Ping An Good Doctor
SATCM: The National Administration of Traditional Chinese Medicine
SC: State Council
